# Polyurea Dendrimer Folate-Targeted Nanodelivery of l-Buthionine Sulfoximine as a Tool to Tackle Ovarian Cancer Chemoresistance

**DOI:** 10.3390/antiox9020133

**Published:** 2020-02-03

**Authors:** Adriana Cruz, Pedro Mota, Cristiano Ramos, Rita F. Pires, Cindy Mendes, José P. Silva, Sofia C. Nunes, Vasco D. B. Bonifácio, Jacinta Serpa

**Affiliations:** 1 iBB-Institute for Bioengineering and Biosciences, Instituto Superior Técnico, Universidade de Lisboa, Avenida Rovisco Pais, 1049-001 Lisboa, Portugal; ab.cruz@campus.fct.unl.pt (A.C.); pedromb.mota@gmail.com (P.M.); ritafpires@tecnico.ulisboa.pt (R.F.P.); 2 CEDOC, Chronic Diseases Research Centre, NOVA Medical School, Faculdade de Ciências Médicas, Universidade NOVA de Lisboa, Campo dos Mártires da Pátria, 130, 1169-056 Lisboa, Portugal; ramos.cristiano.93@gmail.com (C.R.); cindymendes8@gmail.com (C.M.); asofianunes5@gmail.com (S.C.N.); 3 Instituto Português de Oncologia de Lisboa Francisco Gentil (IPOLFG), Rua Prof Lima Basto, 1099-023 Lisboa, Portugal; 4 Hospital Santo António dos Capuchos, Centro Hospitalar Lisboa Central, Alameda Santo António dos Capuchos, 1169-050 Lisboa, Portugal; jospais@gmail.com

**Keywords:** ovarian cancer, chemoresistance, l-buthionine sulfoximine, polyurea dendrimers, carboplatin, nanoparticles-mediated chemotherapy, chemosensitization

## Abstract

Ovarian cancer is a highly lethal disease, mainly due to chemoresistance. Our previous studies on metabolic remodeling in ovarian cancer have supported that the reliance on glutathione (GSH) bioavailability is a main adaptive metabolic mechanism, also accounting for chemoresistance to conventional therapy based on platinum salts. In this study, we tested the effects of the in vitro inhibition of GSH synthesis on the restoration of ovarian cancer cells sensitivity to carboplatin. GSH synthesis was inhibited by exposing cells to l-buthionine sulfoximine (l-BSO), an inhibitor of γ-glutamylcysteine ligase (GCL). Given the systemic toxicity of l-BSO, we developed a new formulation using polyurea (PURE) dendrimers nanoparticles (l-BSO@PURE_G4_-FA_2_), targeting l-BSO delivery in a folate functionalized nanoparticle.

## 1. Introduction

Ovarian cancer is a highly mortal gynecologic cancer [1,2,3], with the late diagnosis and acquired chemoresistance to the conventional therapy as the major hurdles to cure this disease [4,5,6]. The conventional therapy of ovarian cancer is mainly based on comprehensive oxidative/alkylating drugs such as carboplatin in combination with taxanes [7,8,9]. The platinum drugs induce cell death through two mechanisms: the establishment of adducts of DNA and proteins, and the generation of reactive oxygen species (ROS) [9,10]. We have previously found that ovarian cancer cells exhibit a great metabolic reliance on cysteine and glutathione (GSH), as these thiols were shown to protect cells under stressful conditions such as hypoxia or carboplatin exposure, hence, also accounting for chemoresistance [11,12,13]. GSH can explain this protective effect given its role as a ROS scavenger and as a pivotal drug detoxifier, allowing cells to evade cell death [14,15,16,17,18]. We have reported that chemoresistance, supported by high GSH levels, can be abrogated upon the exposure to l-buthionine sulfoximine (l-BSO) [11], an inhibitor of γ-glutamylcysteine ligase (GCL), the first enzyme catalyzing the synthesis of GSH [19,20]. However, the systemic administration of l-BSO is associated to an elevated toxicity, since l-BSO is an irreversible inhibitor of GCL [19]. Therefore, an appropriate approach to revert this systemic toxicity would be the targeted delivery of l-BSO to cancer cells using a nanoparticles-mediated strategy. In this sense, dendrimers are an outstanding drug delivery platform, and many efforts have been made in the last few years to circumvent their cytotoxicity [21], encouraging the fast translation into clinic applications in a near future. In this study, we developed polyurea (PURE) dendrimers, which are a special case of non-toxic [22] and non-hemolytic dendrimers up to very high concentrations (circa 50 μM, unpublished data), and that have already been explored in different nanotherapeutic strategies [23,24,25,26]. The use of folate-targeted dendrimers is an emergent approach in cancer-targeted chemotherapy [27]. As we recently reported [28], folate-targeted polyurea dendrimer generation four (PURE_G4_-FA_2_) nanoparticles are a suitable drug delivery system. Of notice, ovarian cancer cells express higher levels of folate receptor α (FA-Rα) than normal cells [29] and FA-Rα targeted therapy is an assay commonly tested in in vitro and in vivo models of ovarian cancer [30]. In here, we hypothesized that PURE_G4_-FA_2_ encapsulated l-BSO (l-BSO@PURE_G4_-FA_2_) is a suitable strategy to tackle carboplatin resistance, restoring ovarian cancer cells sensitivity to chemotherapy.

## 2. Materials and Methods

### 2.1. Cell Culture 

Two human ovarian cancer cell lines were used, a serous carcinoma (OSC) cell line (OVCAR3-HTB-161™) and a clear cell carcinoma (OCCC) cell line (ES2-CRL-1978™). In order to analyze the effect of nanoparticles in squamous cells, since ovarian cancer metastasizes preferentially to peritoneum, an immortalized squamous epithelium cell line (HaCaT-PCS-200-011™) was also tested. All the cell lines were obtained from American Type Culture Collection (ATCC) (Manassas, VA, USA) and were cultured in Dulbecco’s Modified Eagel Medium (DMEM; 41965-039, Gibco, Life Technologies; Massachusetts, MA, USA). All culture media were supplemented with 10% fetal bovine serum (FBS; S 0615, Merck; Darmstadt, Germany), 1% Antibiotic-Antimycotic (AA; P06-07300, PAN Biotech; Aidenbach, Germany) and 50 µg/mL gentamicin (15750-060, Gibco, Life Technologies; Massachusetts, MA, USA). Cells were maintained in a humidified environment of 5% CO_2_ at 37 °C, until reaching approximately 75–100% optical confluence. Cells were detached with 0.05% Trypsin-EDTA (25300-054, Invitrogen, Thermo Fisher Scientific; Massachusetts, MA, USA) at room temperature (RT) for approximately 5 min, and split to new plates according to the experimental procedures.

### 2.2. Folate Receptor α (FA-Rα) Immunofluorescence 

Sections of 4% paraformaldehyde fixed and paraffin embedded cells (cyto-blocks) were used, after deparaffinization in xylol and re-hydration in a sequence of 5 min incubations in 100%, 70% and 40% ethanol, followed by 10 min in water. Slides were then incubated with the mouse-anti-human FA-Rα (IPI3005G10, BioCare Medical; Pacheco, CA, USA) for 3 h at RT; followed by incubation with the rabbit-anti-mouse Alexa Fluor^®^ 488-conjugated secondary antibody (A-28175, Invitrogen; Massachusetts, MA, USA), 1 h, in the dark, at RT. Slides were mounted in VECTASHIELD media containing DAPI (4′-6-diamidino-2-phenylindole) to counterstain cell nuclei in blue (H-1200, Vector Labs; Burlingame, CA, USA). The analysis was performed by standard fluorescence microscopy in an Axio Imager.Z1 microscope (Zeiss, Oberkochen, Germany). Images were acquired and processed with *CytoVision* software (Leica, Wetzlar, Germany).

### 2.3. l-BSO and FL Encapsulation 

Polyurea (PURE) dendrimers were synthesized following our supercritical-assisted polymerization methodology [22]. FA-NHS and PURE_G4_-FA_2_ were synthesized following our reported protocol [31]. All chemicals and solvents were used as received without further purification. Folic acid (FA) and *N*,*N*-dicyclohexylcarbodiimide (DCC; A10973) (99% purity) were obtained from Alfa Aesar (Kandel, Germany). Fluorescein (FL; F7505), Triethylamine (TEA; 471283) (≥99.5% purity), *N*-hydroxysuccinimide (NHS; 130672) (98% Purity) and L-buthionine sulfoximine (l-BSO: B2515) (≥97% purity) were obtained from Sigma-Aldrich (Darmstadt, Germany).

The encapsulation of FL in PURE_G4_-FA_2_ (FL@PURE_G4_-FA_2_) followed the same methodology used for L-BSO encapsulation [31]. Typically, in a vial, FL (0.0131 mmol, 5.3 mg) was dissolved in 1 mL of distilled water. To this solution, the folate-target dendrimer (PURE_G4_-FA_2_) (6.46 µmol, 56.6 mg) was added. The mixture was then left overnight at RT, in the dark, and under stirring. After this period, the product was purified by dialysis (MWCO 100–500 Da) and characterized by ^1^H NMR.

### 2.4. Confirmation of Cellular Internalization of Nanoparticles by Flow Cytometry

Cells (1 × 10^5^ cells/well) were cultured overnight on 24-well plates and then incubated with several concentrations of FL@PURE_G4_-FA_2_ (0.001–0.120 µM) for 24 h. Only viable adherent cells were collected for the analysis. Cells were then washed with PBS (1×) and detached with trypsin-EDTA. After collection to 1.5 mL Eppendorfs, cells were harvested by centrifugation at 255× *g* for 3 min and washed twice with PBS (1×). Afterwards, cells were suspended in 200 µL of PBS (1×) and samples were analyzed by flow cytometry (FACScalibur–Becton Dickinson; New Jersey, NJ, USA). Sample data was analyzed using *FlowJo* 8.7 software (https://www.flowjo.com). The assay was performed at least in three biological replicates.

### 2.5. Confirmation of Cellular Internalization of Nanoparticles by Fluorescence Microscopy 

The cell lines OVCAR3, ES2 and HaCaT were cultured on glass slides coated with 0.2% gelatine and then incubated with free FL or FL@PURE_G4_-FA_2,_ for 8 and 24 h. After incubation, cells were fixed in 2% paraformaldehyde for 15 min at RT and washed with PBS (1×). The slides were mounted in VECTASHIELD media with DAPI and examined by standard fluorescence microscopy using an Axio Imager.Z1 microscope. The images were acquired with the *CytoVision* software. The assay was performed at least in three biological replicates.

### 2.6. Cell Death Analysis by Flow Cytometry 

The cells (1 × 10^5^ cells/well) were seeded in 24-well plates and cultured overnight in control conditions. The effect of different concentrations of free l-BSO (between 0.05 and 120 mM) and l-BSO@PURE_G4_-FA_2_ (between 3 and 2522 µM) in cell viability was tested for 24 h of exposure. To evaluate the sensitization effect of L-BSO to carboplatin, OVCAR3 cells were exposed to the previous culture conditions combined with carboplatin (25 μg/mL).

After experimental conditions, the detached cells in supernatants were collected, and adherent cells were harvested with 0.05% Trypsin-EDTA. Cells in the supernatant and trypsinized cells were harvested together by centrifugation, 255× *g* for 2 min. Cells were stained with 0.5 μL annexin V-fluorescein (FITC)-(640906, BioLegend, San Diego, CA, USA), in 1× annexin V binding buffer (10 mM HEPES—pH 7.4, 150 mM NaCl, 2.5 mM CaCl_2_, prepared in 1× PBS—pH 7.4), and incubated at RT, in the dark, for 15 min. Samples were resuspended in 200 μL PBS (1×) plus 1% BSA and centrifuged at 255× *g* for 2 min. Cells were resuspended in 200 μL of annexin V binding buffer 1× and 2.5 μL of propidium iodide (PI, 50 μg/mL; P4170, Sigma-Aldrich; Darmstadt, Germany) was added 5 min prior to analysis. Afterwards, samples were analyzed by flow cytometry (FACScalibur–Becton Dickinson; Franklin Lakes, NJ, USA). Data were analyzed using *FlowJo* 8.7 software (https://www.flowjo.com). The assay was performed at least in three biological replicates.

### 2.7. Statistical Analysis 

Statistical analyses were performed in *GraphPad Prism* 7.0 software (www.graphpad.com). Data is presented as mean ± SD. Assays were performed with at least three biological replicates. For comparisons of two groups, two-tailed unpaired *t*-test was used. To compare more than two groups, one-way and two-way analysis of variance (ANOVA) with Dunnets multiple-comparisons test were used. Statistical significance was established at *p* < 0.05; * *p* < 0.05, ** *p* < 0.01, *** *p* < 0.001, **** *p* < 0.0001.

## 3. Results

### 3.1. Ovarian Cancer Cells Internalize PURE_G4_-FA_2_ Nanoparticles in a Dose Dependent Manner

**Figure 1:**
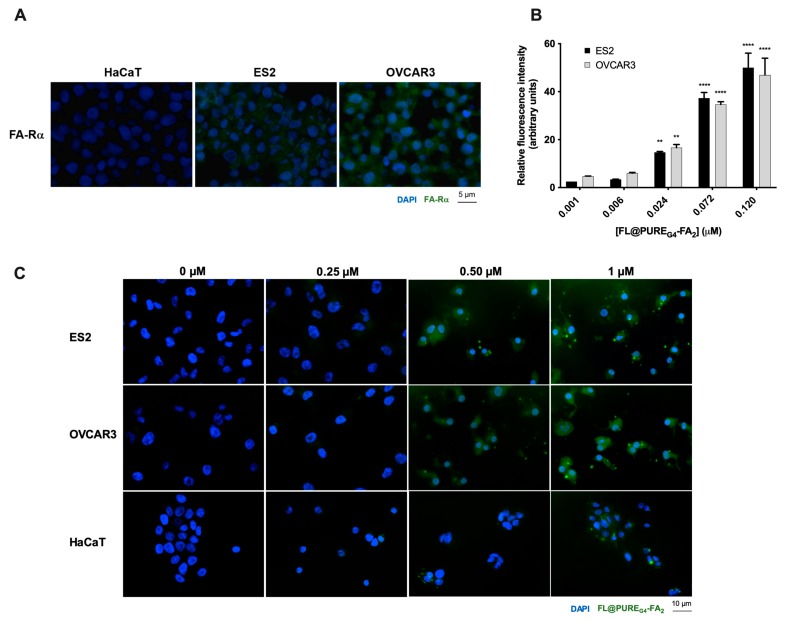

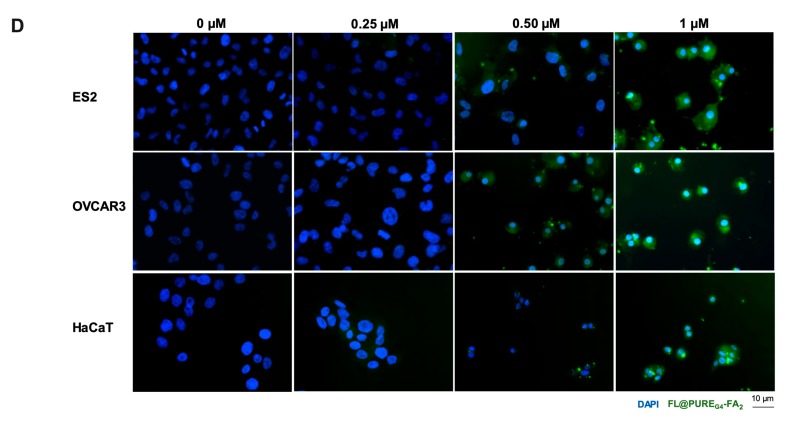
Ovarian cancer cells internalize FL@PURE_G4_-FA_2_ nanoparticles in a dose dependent manner. The expression of folate receptor α (FA-Rα) was evaluated by immunofluorescence in HaCaT, ES2 and OVCAR3 cells (**A**). Ovarian cancer cells (OVCAR3 and ES2) were exposed to different concentrations of PURE_G4_-FA_2_ nanoparticles loaded with fluorescein (FL@PURE_G4_-FA_2_) and the cellular internalization was confirmed by: (**B**) FL fluorescence detected by flow cytometry (after 24 h of incubation); and by fluorescence microscopy (**C**) after 8 h and (**D**) 24 h of incubation. HaCaT cells were used as a control in fluorescence microscopy. In immunofluorescence, nuclei were DAPI (4′,6-diamidino-2-phenylindole) counterstained. Results are shown as mean ± SD. ** *p* ≤ 0.01  **** *p* ≤ 0.0001.

The expression of FA-Rα was confirmed in ovarian cancer (ES2 and OVCAR3) and squamous non-cancer (HaCaT) cell lines. As seen, HaCaT cell are negative for FA-Rα, whereas ES2 and OVCAR3 cells express FA-Rα (Figure 1A). In order to validate the specificity of the internalization of PURE_G4_-FA_2_ by ovarian cancer cells, we tested fluorescein loaded PURE_G4_-FA_2_ (FL@PURE_G4_-FA_2_) prior to test L-BSO@PURE_G4_-FA_2_. By flow cytometry and fluorescence microscopy, we verified that FL is delivered in a dose dependent manner to both ES2 and OVCAR3 cell lines (Figure 1). In HaCaT cells, the internalization of fluorescein was only verified at the highest concentration (1 µM) of FL@PURE_G4_-FA_2_, after 8 and 24 h (Figure 1C,D). This observation supports the affinity of FL@PURE_G4_-FA_2_ to ovarian cancer cells.

### 3.2. l-BSO@PURE_G4_-FA_2_ is More Effective in Inducing Cell Death in Ovarian Cancer Cells than Free l-BSO

**Figure 2:**
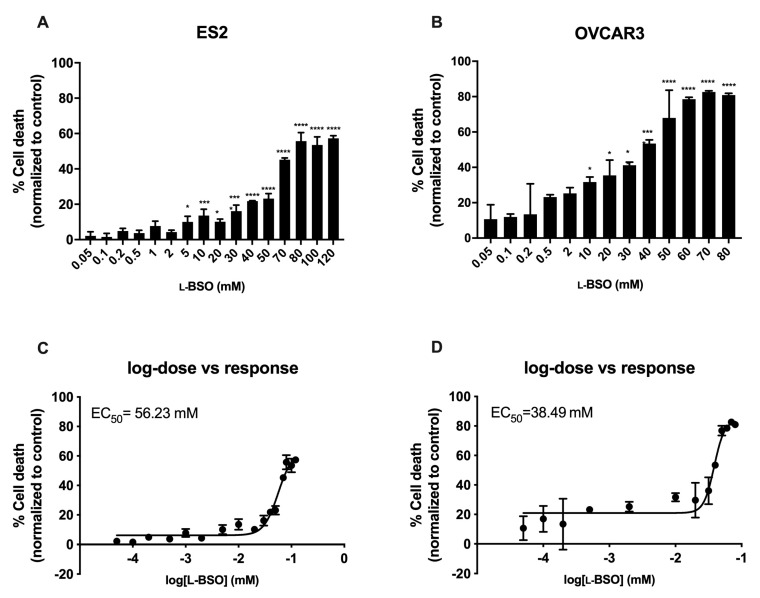
Free L-BSO induces cell death in ovarian cancer cells in a dose dependent manner in a mM scale. Ovarian cancer cells (OVCAR3 and ES2) were exposed to different concentrations of L-BSO and cell death was determined by flow cytometry using annexin V-FITC and propidium iodide (PI) staining. Cell death percentage related to ES2 cells (**A**) and OVCAR3 cells (**B**) is presented. The concentration of L-BSO needed to reach 50% of the maximum cytotoxic effect (EC_50_) was also calculated: (**C**) EC_50_ related to ES2 cells and (**D**) EC_50_ related to OVCAR3 cells. Results are shown as mean ± SD.  * *p* ≤ 0.05 *** *p* ≤ 0.001 **** *p* ≤ 0.0001.

**Figure 3:**
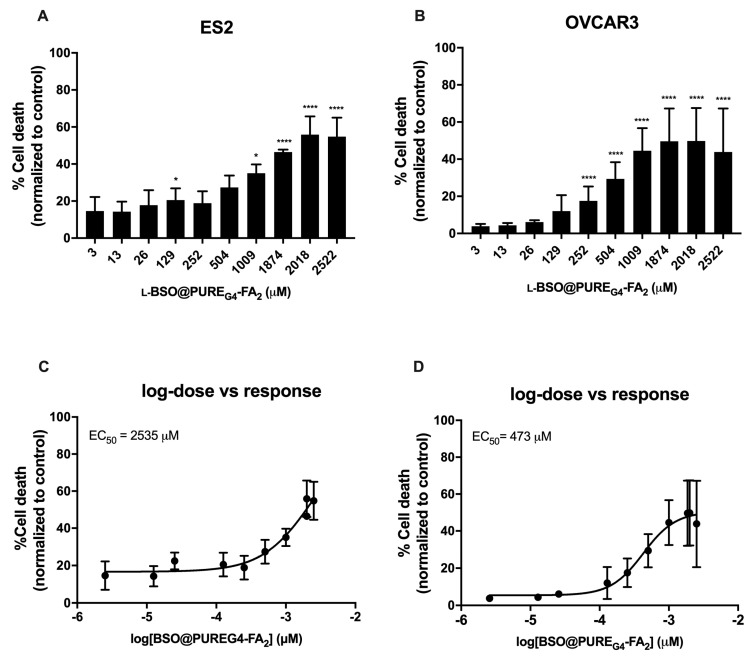
L-BSO@PURE_G4_-FA_2_ induces cell death in ovarian cancer cells in a dose dependent manner in a µM scale. Ovarian cancer cells (OVCAR3 and ES2) were exposed to different concentrations of L-BSO loaded into PURE_G4_-FA_2_ nanoparticles (L-BSO@PURE_G4_-FA_2_) and cell death was determined by flow cytometry using annexin V-FITC and propidium iodide (PI) staining. Cell death percentage related to ES2 cells (**A**) and OVCAR3 cells (**B**) is presented. The concentration of L-BSO@PURE_G4_-FA_2_ needed to reach 50% of the maximum cytotoxic effect (EC_50_) was also calculated: (**C**) EC_50_ related to ES2 cells and (**D**) EC_50_ related to OVCAR3 cells. Results are shown as mean ± SD.  * *p* ≤ 0.05, **** *p* ≤ 0.0001.

The efficacy of inducing cell death by free and encapsulated l-BSO was tested in ovarian cancer cells, in order to verify the advantages of using a l-BSO targeted delivery. In ovarian cancer cells, the concentration needed to reach 50% of the maximum cytotoxic effect (EC_50_) was higher in free L-BSO than in l-BSO@PURE_G4_-FA_2_, indicating a more effective delivery of l-BSO by the l-BSO@PURE_G4_-FA_2_ nanoformulation in comparison to free l-BSO applied directly to the culture media (Figure 2 and Figure 3). The EC_50_ of L-BSO@PURE_G4_-FA_2_ was 22-fold (ES2) and 81-fold (OVCAR3) lower than the EC_50_ of free l-BSO (Figure 2C,D and Figure 3C,D). Furthermore, ES2 cells revealed to be more resistant to the l-BSO effect than OVCAR3, given the higher EC_50_ for L-BSO@PURE_G4_-FA_2_ and l-BSO in this cell line compared to OVCAR3 (Figure 2C,D and Figure 3C,D).

### 3.3. l-BSO@PURE_G4_-FA_2_ Is More Cytotoxic to Ovarian Cancer Cells than to Non-Cancer Squamous Cells

**Figure 4:**
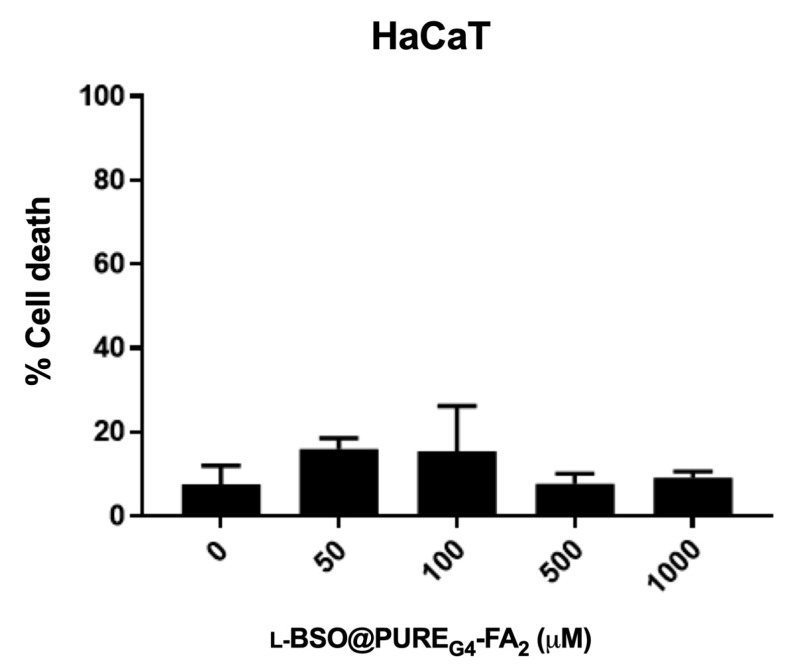
L-BSO@PURE_G4_-FA_2_ does not induce cell death in non-cancer squamous cells (HaCaT). HaCaT cells were exposed to different concentrations of l-BSO loaded into PURE_G4_-FA_2_ nanoparticles (L-BSO@PURE_G4_-FA_2_) and cell death percentage was determined by flow cytometry using annexin V-FITC and propidium iodide (PI) staining. Results are shown as mean ± SD.

l-BSO@PURE_G4_-FA_2_ was also tested in non-cancer squamous cells, as an attempt to address the effect in the peritoneal squamous cells, trying to anticipate a future therapy applied by intra-abdominal infusion. Thus, the cell death levels in HaCaT cells were evaluated upon exposure to l-BSO@PURE_G4_-FA_2_, and no differences were observed between cells exposed to different concentrations of l-BSO@PURE_G4_-FA_2_ (Figure 4). Interestingly, the highest concentration tested (1000 µM) induced about 10% of cell death in squamous cells, whereas the same concentration induced more than 40% of cell death in ovarian cancer cells (Figure 3A,B and Figure 4). This result supports that l-BSO targeted delivery can be a good strategy to treat ovarian cancer without strongly affecting non-cancer cells, at least in a re-sensitizing therapeutic protocol to overcome resistance to platinum salts.

### 3.4. l-BSO@PURE_G4_-FA_2_ Is Effective in Increasing the Sensitivity of Ovarian Cancer Cells to Carboplatin

**Figure 5:**
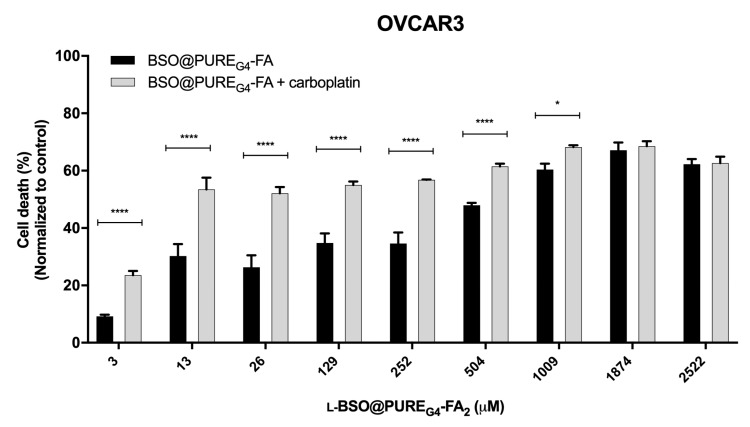
L-BSO@PURE_G4_-FA_2_ sensitizes ovarian cancer cells to carboplatin toxicity. Ovarian cancer cells (OVCAR3) were exposed to increased concentrations of L-BSO@PURE_G4_-FA_2_ with and without carboplatin (25 µg/mL). Cell death was determined by flow cytometry using annexin V-FITC and propidium iodide (PI) staining. Results are shown as mean ± SD. * *p* ≤ 0.05 **** *p* ≤ 0.0001.

To validate our re-sensitizing approach, the OVCAR3 ovarian cancer cell line was exposed to increasing concentrations of L-BSO@PURE_G4_-FA_2_ separately or combined with carboplatin. Overall, l-BSO@PURE_G4_-FA_2_ exposure improved the cytotoxic effect of carboplatin. Furthermore, l-BSO@PURE_G4_-FA_2_ by itself increased cell death, showing again the reliance of ovarian cancer cells on GSH bioavailability (Figure 5). However, the highest concentrations of l-BSO@PURE_G4_-FA_2_ did not improve carboplatin cytotoxicity, which can be related to the threshold of cell capacity of internalizing nanoparticles.

## 4. Discussion

**Figure 6:**
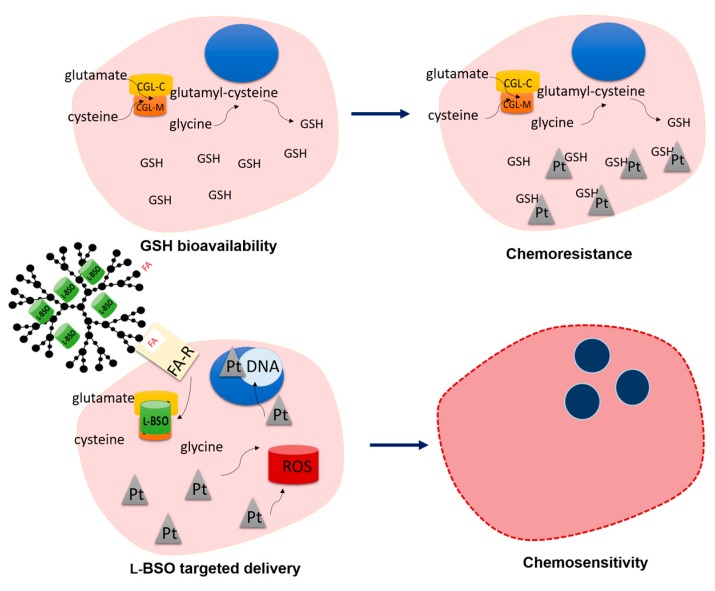
Rational of l-BSO@PURE_G4_-FA_2_ sensitization to carboplatin toxicity in chemoresistant cancer cells. Cancer cells presenting a high glutathione (GSH) bioavailability are commonly resistant to platinum salts (Pt) toxicity, since GSH is a reactive oxygen species (ROS) scavenger and a xenobiotic detoxifying system. l-Buthionine sulfoximine (l-BSO) is an irreversible inhibitor of α-glutamylcysteine ligase (GCL; which has catalytic and modulator subunits, GCLC and GCLM) responsible for GSH synthesis. The targeted delivery of L-BSO in folate-functionalized polyurea dendrimer generation four (PURE_G4_-FA_2_) nanoparticles, taking advantage of the increased expression of FA-Rα in cancer cells, will be efficiently internalized, inhibiting the synthesis of GSH. Therefore, carboplatin will act through its mechanisms of action, ROS generation and adducts formation, culminating in cancer cells death.

Acquired chemoresistance is a critical issue in oncology and ovarian cancer is a paradigm of this matter. Therefore, the development of strategies to overcome chemoresistance is required for a more effective treatment of ovarian cancer [8,15,32,33]. Following our insights on ovarian cancer metabolic remodeling and therapy response [11,12,13], we posited that a FA-Rα-targeted delivery of l-BSO can be a promising strategy to revoke resistance to carboplatin (Figure 6).

We have previously shown the efficacy of PURE_G4_-FA_2_ nanoparticles as a vehicle to deliver a cytotoxic selenium–chrysin compound to ovarian cancer cells [28]. In this study, we have shown the efficacy of those nanoparticles as a vehicle to delivery also l-BSO to ovarian cancer cells. By using fluorescein loaded PURE_G4_-FA_2_, we verified that ovarian cancer cells are more competent in the internalization of these nanoparticles when compared to non-cancer cells. The observed fluorescein uptake by non-cancer squamous cells at the highest tested concentration can be explained by the use of static cultures and by the fact that after 24 h some particles can adsorb to the cells in a nonspecific way. Nevertheless, our results confirmed that the high levels of FA-Rα expression by cancer cells [29] can be explored as a way to reduce the effect of l-BSO in non-cancerous cells. Indeed, this fact allows a preservative systemic therapeutic approach, since l-BSO also induces GSH depletion [34,35] in normal cells, thus, rendering L-BSO otherwise too toxic for therapy. In the early 1990s, L-BSO was used as a drug to treat cancer [36,37], but its adverse effects were so severe that its use was promptly interrupted. However, more recently, L-BSO has regained attention, and several studies reported the use of this compound in cancer preclinical models [38,39,40,41,42].

**Figure 7:**
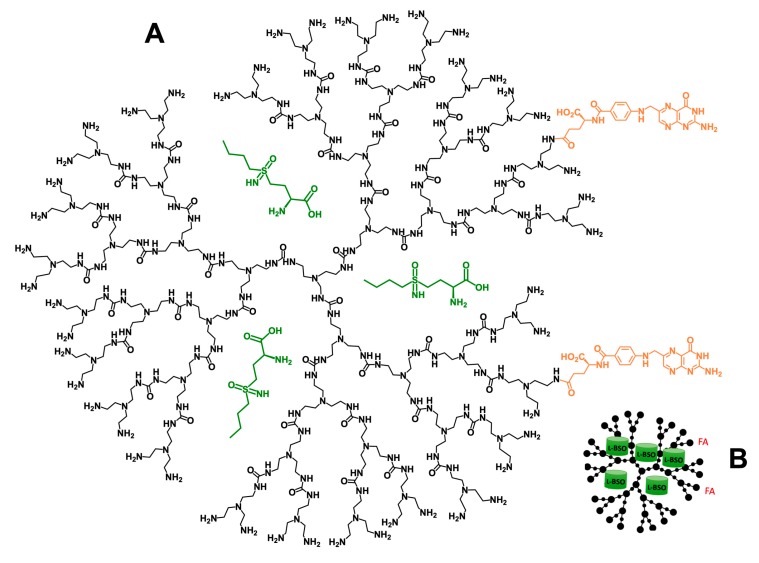
l-BSO@PURE_G4_-FA_2_ nanoformulation. Chemical (**A**) and cartoon (**B**) representation of the nanoformulation. L-BSO (green color) is encapsulated in a folate-targeted (orange color) generation four polyurea dendrimer (PURE_G4_-FA_2_).

Prior to this study, we demonstrated that free l-BSO efficiently diminishes GSH bioavailability, impairing resistance to carboplatin [11]. Importantly, this effect of L-BSO was also observed in an in vivo model of ovarian cancer, reducing significantly subcutaneous tumor size and GSH levels, as well as peritoneal dissemination [11]. In the present study, we verified that a l-BSO@PURE_G4_-FA_2_ nanoformulation (Figure 7) is more effective in inducing ovarian cancer cells death than free l-BSO; and that ovarian cancer cells are more sensitive to l-BSO@PURE_G4_-FA_2_ than non-cancer squamous cells (HaCaT), reinforcing a putative therapy mediated by abdominal infusion.

Our previous studies suggested a stronger dependence of ES2 cells on GSH turnover compared with OVCAR3 cells [12,13], which was also evidenced in this study, as higher EC_50_ of free l-BSO and l-BSO@PURE_G4_-FA_2_ were determined for ES2 compared with OVCAR3 cells. Furthermore, concerning resistance to carboplatin, we have reported that upon carboplatin exposure ES2 produce higher levels of GSH [11] together with an accelerated GSH turnover, compared with OVCAR3 [28]. Therefore, our greatest achievements in this study were the effective use of l-BSO@PURE_G4_-FA_2_ nanoparticles to the specific targeting of malignant cells, decreasing the harmful effects of l-BSO in non-malignant cells, and the similar effective targeting of ovarian cancer cells with different levels of chemoresistance. Together, our study supports the use of l-BSO@PURE_G4_-FA_2_ nanoparticles as a powerful strategy for ovarian cancer treatment.

## 5. Conclusions

More validation studies, namely *in vivo* assays, aiming to evaluate the systemic cytotoxic effect of l-BSO@PURE_G4_-FA_2_, are needed. Nevertheless, our study points out this new nanoformulation as a way of avoiding l-BSO systemic toxicity, and as a tool to abolish cancer cells resistance to carboplatin or putatively to other alkylating/oxidative drugs. In the future, this approach may be applied to other chemoresistant cancers.
